# Motivations and Barriers for the Use of Face Coverings during the COVID-19 Pandemic: Messaging Insights from Focus Groups

**DOI:** 10.3390/ijerph17249298

**Published:** 2020-12-12

**Authors:** Victoria S. Shelus, Simone C. Frank, Allison J. Lazard, Isabella C. A. Higgins, Marlyn Pulido, Ana Paula C. Richter, Sara M. Vandegrift, Rhyan N. Vereen, Kurt M. Ribisl, Marissa G. Hall

**Affiliations:** 1 Department of Health Behavior, Gillings School of Global Public Health, University of North Carolina at Chapel Hill, 170 Rosenau Hall, CB #7400, 135 Dauer Drive, Chapel Hill, NC 27599, USA; vshelus@unc.edu (V.S.S.); simone_frank@med.unc.edu (S.C.F.); marlyn@live.unc.edu (M.P.); apcr@live.unc.edu (A.P.C.R.); sara.vandegrift@unc.edu (S.M.V.); kurt_ribisl@unc.edu (K.M.R.); mghall@unc.edu (M.G.H.); 2 Carolina Population Center, 123 West Franklin St., Suite 210, Chapel Hill, NC 27516, USA; ihiggins@email.unc.edu; 3 Hussman School of Journalism and Media, University of North Carolina at Chapel Hill, 117 Carroll Hall CB#3365, Chapel Hill, NC 27599, USA; rhyan.vereen@unc.edu; 4 Lineberger Comprehensive Cancer Center, CB#7295, University of North Carolina at Chapel Hill, Chapel Hill, NC 27599, USA

**Keywords:** COVID-19, health communication, face coverings, masks, health behavior

## Abstract

Widespread use of face coverings is a key public health strategy to prevent the spread of COVID-19. However, few studies have examined why Americans use or do not use face coverings, and little is known about the most effective messaging strategies. This study explored perceptions of face coverings, including motivations and barriers for use, and examined reactions to messaging promoting the use of face coverings. Six virtual focus groups were conducted with 34 North Carolina residents in July 2020. Participants reported high compliance with face covering recommendations but often did not wear them around family, friends, and colleagues. The most prevalent motivation for the use of face coverings was to protect or respect other people, including high-risk populations and individuals. Other motivators were self-protection, responsibility, desire for control, requirements, and expert advice. Barriers included physical and social discomfort, confusion or misinformation, low perceived susceptibility to COVID-19, and perceptions of identity and autonomy. Even among individuals who frequently wear face coverings, there are opportunities to improve compliance. Messaging should highlight how face coverings protect the wearer and others around them, normalize the use of face coverings in social settings, and emphasize requirements. Positive messages that focus on unity, personal experiences and the rationale for face coverings are recommended.

## 1. Introduction

As of early December 2020, over 14 million confirmed cases and 276,000 deaths in the United States have been attributed to the novel coronavirus disease 2019 (COVID-19) pandemic [1]. The Centers for Disease Control and Prevention (CDC) and White House Coronavirus Task Force recommend individuals wear face coverings in public settings to mitigate COVID-19 transmission, particularly when physical distancing is difficult [2]. However, few studies have examined why individuals use or do not use face coverings, and little is known about messaging strategies for encouraging the public to adopt this behavior.

Recent studies on face coverings have focused primarily on their efficacy to prevent the spread of COVID-19 [3,4,5,6] and public compliance with recommendations [7,8,9]. While recent polls suggest a majority of Americans wear face coverings in public most of the time, few wear them all of the time, as recommended by the CDC and other health officials [9]. To promote proper and consistent face covering use, it is critical to examine attitudes, beliefs, and motivations associated with face coverings. Additionally, it is important to evaluate messaging strategies to improve face covering use, so messaging can have the intended effect of encouraging individuals to comply with public health recommendations [10]. While some studies have explored perceptions of COVID-19 mitigation strategies and general public health guidance within the United States [11,12,13], few studies have examined the public’s attitudes toward face coverings [8,14] or communication strategies to promote their use [15,16].

In this study, we aimed to qualitatively explore perceptions of face coverings and to examine reactions to messaging intended to promote the use of face coverings. This paper will describe the use of face coverings, motivations for wearing face coverings, reasons for not using face coverings, and insights for messaging strategies derived from our study population.

## 2. Materials and Methods

This study was conducted in North Carolina (NC) in July 2020. As of 1 July, there were over 66,500 COVID-19 cases and 1300 deaths reported across the state. Amid worsening disease indicators and increased evidence on the effectiveness of face coverings, Governor Roy Cooper issued a statewide executive order requiring the use of face coverings in public spaces for all individuals over two years of age on 24 June [17]. Previous guidance strongly recommended the use of face coverings.

Focus group participants were recruited through the NC Department of Health and Human Services’ (NC DHHS) community networks using email and social media platforms. Individuals interested in participating in a focus group contacted the study team via email or text message and completed a brief online screening questionnaire in English or Spanish to confirm eligibility. Based on NC DHHS input, we purposively sampled adults, aged 18 and older, living in NC and identifying as (1) Latina/o/x, (2) Black or African American, (3) white and living in a rural area, or (4) young adult (ages 18–25). A short demographic survey and written informed consent were obtained electronically from all participants. The study was approved by the University of North Carolina Office of Human Research Ethics/Institutional Review Board (protocol #20-0995).

Six semi-structured, virtual focus group discussions were conducted with 34 participants between 6 and 9 July 2020. Focus groups were hosted through the online videoconferencing software Zoom (https://zoom.us/) and lasted approximately 90 min. Each focus group included 5–7 participants from one of our target demographic groups: Latina/o/x (two groups; one English-speaking, one Spanish-speaking), Black or African American (one group), white and living in a rural area (one group), and young adults ages 18–25 (two groups; one Black, Indigenous, and people of color (BIPOC), one white). All focus groups were audio-recorded, translated (if conducted in Spanish), and transcribed.

Each focus group included a facilitator, note-taker, and technical support contact from the study team. Facilitators led the groups using a semi-structured discussion guide with questions and suggested probes (see Supplementary Materials) but often used spontaneous probes to encourage elaboration or elicit additional detail from participants. The initial discussion centered around behaviors, motivations, and barriers related to the use of face coverings. Participants were asked to describe the situations in which they wore or did not wear face coverings, as well as the reasons for that behavior. The discussion also explored the reasons other people in their communities, including participants’ family and friends, did not wear face coverings. The second half of the focus group focused on public health messaging, including memorable messages on face coverings and reactions to sample messages developed by the study team promoting the use of face coverings (Table S1).

Qualitative thematic content analysis techniques were used to analyze the data, following a process of reading, coding, data display, and reduction [18]. All transcripts were read and coded based on questions in the focus group discussion guide and emerging themes using the qualitative data analysis software NVivo 12 (QSR International) by one member of the study team. Primary coding reports on behaviors, motivations, barriers, and messaging insights related to face coverings were extracted and further analyzed. Emergent sub-themes were codified and applied to data in coding reports. Memos were developed to summarize findings within each broad theme. Members of the study team who participated in the focus group discussions reviewed and discussed all memos to ensure the key themes and subthemes from each group were accurately represented.

## 3. Results

Among 34 focus group participants, the average age in the two young adult groups was 21 years, while the average age among the remaining four focus groups with adults was 43 years (Table 1). Our sample was primarily female (82%), with 41% identifying as Black or African American and 44% identifying as Latinx. Almost all participants reported wearing face coverings most or all of the time in the demographic survey (91%); however, notable instances where compliance could be improved were shared in the focus group discussions.

### 3.1. Use of Face Coverings

Participants reported high compliance with statewide recommendations on the use of face coverings in public, indoor settings where maintaining distance from others is impossible or impractical, including while grocery shopping, picking up take-out food, attending religious services, or during medical appointments. Participants did not wear face coverings in situations where they were alone, with household members, or while outside and able to social distance (e.g., while driving, at home, or exercising outdoors). The use of face coverings around family, friends, and colleagues varied depending on the context. Many participants observed incorrect use of face coverings in their communities, specifically wearing face coverings below the nose or on the chin.

### 3.2. Motivations for Wearing Face Coverings

#### 3.2.1. Protection

Across groups, the most prevalent motivation for the use of face coverings was to protect or respect other people (Table 2). While many spoke generally about others, some specified the protection of high-risk populations or family members, such as elderly relatives, children with asthma, and infants. Many participants feared giving someone COVID-19. In the words of a rural white participant, *“I wear it more so for protection of others…I would rather wear a mask for a few minutes…than risk not showing symptoms and get somebody else sick. It’s a small price to pay, just to protect other people.”*

#### 3.2.2. Community Responsibility and Norms

Face coverings were often worn out of a sense of responsibility or because of the perception that it was the right thing to do. While most participants suggested their decision to wear a face covering was not affected by the behavior of others around them, some wore face coverings out of a desire to “keep the peace” by following community norms.

#### 3.2.3. Fear, Anxiety, and Controlling the Situation

The sense of security or control provided by wearing a face covering was a motivator, especially in situations where others were not compliant with recommendations. Participants across several groups expressed feeling upset in the presence of others not wearing face coverings, and a heightened sense of anxiety was especially prevalent in Latinx focus groups.

#### 3.2.4. Compliance and Recommendations

Requirements to wear face coverings, typically at the business level, were mentioned across all groups. Participants expressed a desire to follow the rules. While most participants wore face coverings prior to mandates, requirements were the primary motivation for a few and served as an important reminder even to those inclined to wear them. Many participants were also motivated to wear face coverings based on the recommendation of health experts and data supporting their efficacy.

### 3.3. Reasons for Not Using Face Coverings

#### 3.3.1. Perceived to be Unnecessary

Participants commonly reported not wearing face coverings in situations where they felt they were unnecessary, such as while outdoors and able to maintain distance from other people (Table 3). Participants reported not wearing face coverings if they were walking around their neighborhood or socializing with others outside.

Around close friends, family members outside the household, and sometimes coworkers, focus group participants often did not wear face coverings. While participants described wearing face coverings in work settings while interacting with restaurant customers or patients in healthcare clinics, employees sometimes interacted without face coverings in office settings or outside public view. Not wearing face coverings with friends and family was often attributed to knowing and trusting the precautions others had taken during the pandemic. An African American participant said, *“It’s just about kind of who it is, and the trust level I have that they’ve been safe,”* describing how the decision about whom to see without face coverings is made.

#### 3.3.2. Difficulty Navigating Social Situations

While most participants appeared comfortable with the decision to forego face coverings around select family and friends, some Latinx Spanish-speaking participants expressed discomfort and found it challenging to navigate social situations. They feared offending others by asking them to wear a face covering or keep their distance and felt unsure about how to broach the subject.

#### 3.3.3. Confusion or Misinformation

Concerns about efficacy and mixed messaging are preventing face coverings from being consistently adopted. At the beginning of the pandemic, the American public was asked to reserve high-quality masks for health care providers. While public health officials later encouraged everyone to wear cloth face coverings, the conflicting messages and news coverage contribute to confusion. As one English-speaking Latinx participant stated, *“Everybody’s getting mixed signals…Some TV shows are saying yes [wear masks], news is saying no [don’t wear masks]…Nobody knows for sure…we’re all just trying to figure it out.”*

Participants also expressed uncertainty about whether face coverings protected the individual wearing them or only others around them. Some endorsed the belief that face coverings would be more widely adopted if individual benefits were clear. While not frequently mentioned, myths and conspiracy theories came up in several groups. Participants described concerns about self-exposure to carbon dioxide while wearing face coverings and beliefs among friends or family that COVID-19 is part of a harmful government agenda or profit scheme.

#### 3.3.4. Physical Challenges

Many participants disliked wearing face coverings and complained about the heat, difficulty breathing, glasses fogging up, difficulty hearing other people, and, among some of the Latinx participants, being understood as a non-native English speaker while wearing one. However, participants overwhelmingly noted they wear face coverings despite their physical discomfort, often stating it was the least they could do. Some participants changed their type of face covering to be more breathable or less irritating on the ears. While forgetting a face covering was another challenge, several participants kept face coverings in various locations to make it easier to remember.

#### 3.3.5. Low Perceived Susceptibility

Some participants had friends or family who believed face coverings were unnecessary, either because they were not sick, did not believe that they were personally at risk, or felt protected by their religious beliefs. Latinx participants mentioned the idea of “si Dios quiera” (loosely translated as “it is in God’s hands”), which communicates a belief that COVID-19 transmission is out of our control.

#### 3.3.6. Identity and Autonomy

In some focus groups, participants shared stories about themselves or family members not wearing face coverings because of concerns about how others would perceive them. Rural white participants believed men in their lives were not wearing face coverings because of their perceptions of masculinity or face coverings as uncool. A Latinx father shared how his teenage son did not want to be the only one wearing a face covering in a social setting. One Spanish-speaking participant felt pressured because of concern about what others might think, saying, *“You feel the pressure….If you don’t go out, it’s like you’re dumb or you live in fear or you’re letting the government control your rights.”*

Perceptions of the efficacy and importance of face coverings may stem from the politicization of their use. A few focus group participants reported knowing people who did not wear face coverings because of their political affiliation or the lack of a strong endorsement from the President of the United States. Wearing face coverings was also viewed as an infringement on individual rights, and even participants who consistently wore face coverings disliked being told what to do.

### 3.4. Insights for Messaging Strategies

#### 3.4.1. Message Tone

When asked about their views on sample messages, focus group participants preferred positive and encouraging messages, rather than messages perceived as negative, threatening, or overly instructive. One Latinx participant questioned, *“What happened to the good things? What happened to you know this 80-year-old lady had it and she recovered? There’s no positivity about anything, and it’s frustrating that all you’re bombarded with all day is negative, negative, death, death…You become insensitive to it.”* Positive messages were especially appreciated by some, given all the negativity in the news.

Face covering messages that promoted unity and diversity and the reminder that “we’re all in this together” were well-received across groups. A Latinx English-speaking participant described how strongly messaging with the word “together” resonates, *“When you say Spanish or English, I think the use of that word conveys that the responsibility is not all on you. It’s a public service that’s being asked of everyone, and burdens are better handled together.”* In addition to togetherness, messaging focused on family, community, faith, and doing it for others appealed to Latinx participants, who often did not feel that current messaging resonated with them.

#### 3.4.2. Message Content

Personal stories from individuals affected by COVID-19 were impactful. Some participants described memorable stories they heard on the news or social media about healthcare workers or family members whose loved ones had died. Others knew people who had tested positive. For example, a white young adult participant shared her family’s experience, *“My sister actually got COVID…And she’s young and healthy, but she was stuck in bed for two weeks straight. And so seeing someone who’s young and healthy getting you know so sick from this, it definitely shaped me and my family’s view.”* Many believed that for those resistant to face coverings, perceptions and behavior were unlikely to change unless someone close to them was affected.

There were conflicting views on messaging about high-risk populations or that encouraged the use of face coverings to reopen businesses. Some believed highlighting older adults or those with pre-existing health conditions could promote a false sense of invincibility for others outside of those groups. Similarly, while some participants felt motivated to use face coverings out of a desire to open-up or resume educational, work, or leisure activities, others, particularly young adults, cautioned against overly optimistic promises about a return to normal.

Short, catchy messages were positively received and believed to be a good reminder to people already wearing face coverings; however, some participants desired more data or rationale in messages. Additionally, some expressed a need for clarification on mixed messages and answers to nuanced questions about the use of face coverings.

## 4. Discussion

As the COVID-19 pandemic continues to affect communities across the United States, it is critical to understand when and why individuals are wearing face coverings. Even among individuals who frequently wear face coverings, there are opportunities to improve compliance, particularly in social settings. Reminders and other messages could increase how often compliant individuals wear face coverings. These insights should inform messaging strategies to encourage the use of face coverings in NC and beyond (Table 4).

Protection, for others and oneself, remains a consistently endorsed motivator for wearing face coverings. As the evidence continues to emerge [6,19,20,21], messaging should highlight how face coverings are our most promising public health strategy to keep ourselves and loved ones safe.

Participants frequently did not wear a face covering around trusted family and friends or while interacting with coworkers in office settings. These interactions were not perceived as occurring in public. These perceptions are problematic, as cluster infections among family, friends, and colleagues remain a common pattern for COVID-19 transmission [22,23]. Promoting the use of face coverings in social and work settings is needed. Additionally, given the role of trust in the decision to wear a face covering, as well as discomfort asking contacts to wear them, messaging strategies should normalize the use of face coverings around family, friends, and coworkers.

Requirements to wear a face covering, especially at businesses and workplaces, should be emphasized. These messages serve as reminders or “cues to action” for those already wearing face coverings [24]. Additionally, requirements may be needed to convince those who are less compliant or resistant to the use of face coverings. Participants often focused on individual business decisions rather than an overarching state-level policy. Moreover, some participants expressed resistance to being told what to do, a phenomenon known as “reactance [25].” Therefore, notices such as “face coverings are required here” may be more acceptable and elicit less reactance than phrasing that could be perceived as taking away freedoms, such as mentioning a “law” or “mandate”.

Successful messages may include personal experiences, data, rationale for the use of face coverings, and clarification on topics of confusion. Similar themes have been suggested promoting other COVID-19 behavioral intervention strategies, such as social distancing [26]. While focusing on high-risk groups and recovery may resonate with some individuals, there could be unintended consequences associated with promoting invincibility or an overly optimistic narrative about the projected trajectory of COVID-19. Given widespread reporting of the incorrect use of face coverings, messaging should provide guidance on correct use.

Finally, participants strongly felt that messages should be framed as positive and encouraging, rather than negative, threatening, or overly instructive. Themes of unity and diversity were desired across all focus groups. Prior studies have shown that messages evoking both negative (e.g., fear) and positive (e.g., hope) emotions can elicit beneficial behavior changes [27,28], so future research should examine these possibilities in the context of messages to promote face coverings. More research into the language that resonates with specific communities is needed, especially considering the inequities in COVID-19 morbidity and mortality among underserved minority populations [29]. In our study, Latinx participants did not believe that current messaging resonated with them. This is especially important given high COVID-19 prevalence and related anxiety among this population, which may be compounded with fear of accessing health services and resources, particularly for undocumented individuals.

### Strengths and Limitations

This study provides important insights into motivations and barriers for the use of face coverings and could help inform health communication strategies to increase their use and prevent the spread of COVID-19. However, further investigation may be needed to confirm whether the motivations and barriers identified among our study population, as well as messaging recommendations, generalize to other settings. Moreover, while we believe we reached saturation with our entire sample, the study was not designed with the goal of achieving saturation within our target demographic groups. Therefore, themes specific to certain groups, but not the entire sample, may not be representative of all individuals within these groups across NC.

Additionally, our conclusions are limited by a convenience sample that was highly compliant with current face covering requirements. Self-selection bias may have resulted in the inclusion of study participants with more positive perceptions and greater use of face coverings than the general population, as well as an overrepresentation of women in our sample. This could be partially attributed to our recruitment through NC DHHS networks. Social desirability bias could also have affected participant responses, as lack of compliance with recommendations could be viewed unfavorably by others. While focus group participants reported the reasons their friends and family do not always wear a face covering, our insights on barriers are often secondhand.

## 5. Conclusions

Understanding motivations and barriers for use are critical to inform health communication and impact health behavior. Among our study sample, protection for others and one’s self is often the driving motivation for the use of face coverings, along with the desire to adhere to requirements, maintain some control, and do the right thing. However, challenges to the consistent and widespread use of face coverings remain as individuals navigate discomfort both physically and in social settings. Communication strategies should be guided by local audience insights to deliver relevant and timely messages to positively encourage preventive behaviors during the pandemic.

## Figures and Tables

**Table 1:** Characteristics of focus group participants.

Characteristic	*n* = 34 Mean (Min–Max) or % (*n*)
Age–young adult	21 years (19–24)
Age–adult	43 years (26–64)
Sex	
Male	15% (5)
Female	82% (28)
Other or prefer not to answer	3% (1)
Race ^1^	
White	47% (16)
Black or African American	41% (14)
Other	15% (5)
Missing	6% (2)
Ethnicity	
Latino/a/x	44% (15)
Non-Latino/a/x	56% (19)
Location	
Urban	29% (10)
Suburban	44% (15)
Rural	26% (9)
Region	
Western NC/Mountains	26% (9)
Central NC/Piedmont	53% (18)
Eastern NC/Coastal Plains	21% (7)
Face covering use	
Never	0% (0)
Some of the time	9% (3)
Most of the time	35% (12)
All of the time	56% (19)

^1^ Participants could select more than one race.

**Table 2:** Motivations for the use of face coverings among North Carolina residents.

Category	Motivation	Example Quote
Protection	To protect others	*“It goes back to people’s…family and their loved ones and why we’re putting on masks, because we want to protect other people, because we care about other people.”* (Latinx English)
	To protect high-risk individuals	*“You don’t know what someone else is like dealing with…Whether they are younger, old, it doesn’t matter. I have a family member that’s asthmatic, I have older family members with other health issues. So I try to wear it out of respect for them.”* (Young adult BIPOC)
	To protect myself	*“I [have] an autoimmune disorder, and I am under extreme immunosuppressants...I do have to wear a face mask for my own safety.”* (Young adult BIPOC)
Community responsibility and norms	Sense of responsibility	*“I feel like it’s important for us to all be role models, and I’ve been wearing it because I think it’s the right thing to do.”* (Rural white)
	To follow community norms	*“If someone’s going to get annoyed if they see me in a store and they’re going to feel unsafe even though I’m healthy, and I’m going to cause some kind of problem, well, I prefer to have the mask, more than anything to keep the peace.”* (Latinx Spanish)
Fear, anxiety, and controlling the situation	For safety or in response to fear	*“[It’s] supposed to be…if you feel any symptoms…then don’t go around people. And some people don’t follow that…So [I wear one] just, I guess, out of partly fear.”* (Latinx English)
	For a sense of control	*“We are in charge at this point of our own safety…I don’t think…we could really depend on our government officials and leadership…and so our PPE [personal protective equipment] is really all we have right now.”* (Black or African American)
Compliance and recommendations	To comply with requirements	*“I wear a mask wherever it’s required to wear a mask. When I go to the store, or if I go to the doctor’s office…If it’s not required, I do not…That’s the only reason I’m wearing it, is because it’s required.”* (Rural white)
	Based on data/experts	*“If the data is saying that it helps to wear it, then I’m going to do what the researchers are saying that we should do.”* (Black or African American)
Reopen business and schools	To resume activities	*“I would love to be able to go back into the office…And so whatever steps I can take myself to get back to that. Like, that’s what I’ll do.”* (Young adult white)

**Table 3:** Reasons for not using face coverings among North Carolina residents.

Category	Reason	Example Quote
Perceived to be unnecessary	Social distancing is possible	*“I’ll go for a walk around the neighborhood…and I’m typically able to stay like far away from people. So I don’t feel like I need to wear one.”* (Young adult white)
	With people they trust	*“In the visits with other family members, we haven’t worn face masks… since they’ve been making wise decisions…and they’ve been saying that they are doing the right things, then we feel comfortable not wearing a mask.”* (Young adult BIPOC)
Difficulty navigating social situations	Fear offending others	*This week we decided, ‘we appreciate your friendship, don’t get offended, but we think it would be better if we didn’t see each other anymore, for now, this closely.’…They got offended.”* (Latinx Spanish)
Confusion or misinformation	Mixed messages	*“People are confused and they’re saying, well, you said it 4 months ago it was okay. We didn’t have to worry about a mask, and now you’re saying we need to wear them…That’s how people are getting mixed messages.”* (Rural white)
	Don’t protect the wearer	*“You also have to remember, the mask doesn’t protect you…It protects other people. And I think that that’s a problem in general. I think you would have a lot more compliance if people thought that the mask was protecting them…because we look to protect ourselves first.”* (Rural white)
	Myths and conspiracy theories	*“I have family members that think that it’s a propaganda for profit. Other theories they have are that it’s just another way for us to have our immunity inhibited. They think there’s a larger harmful agenda behind the scenes.”* (Latinx English)
Physical challenges	Uncomfortable	*“It’s very uncomfortable…it’s very hot, but I’ll pull it off or away from my face for a couple of seconds. And I’ll put it right back on.”* (Black or African American)
	Respiratory issues	*“I haven’t been running or exercising in a while, but like when I did…I would just keep my distance most of the time, because like I have asthma. So if I were running like I feel like the mask would bother me.”* (Young adult BIPOC)
	Forgot a face covering	*“Sometimes they say that they forgot to bring a face mask or like they don’t have one.”* (Young adult BIPOC)
Low perceived susceptibility	Not sick with COVID-19	*“A friend who wanted to come help me…she took off her mask, and she said, “Take off your mask, there’s no problem, I’m healthy.”* (Latinx Spanish)
	Believe it wouldn’t happen to them	*“A lot of people maybe still have the mentality of, well, it won’t happen to me or like, you know, I couldn’t imagine that happening in my family.”* (Rural white)
	Religious or spiritual beliefs	*“I’ve also heard a few people say based on their religious beliefs, who’ve said well ‘God will protect me. You know, I’m safe, he’s protecting me.’”* (Rural white)
Identity and autonomy	Peer pressure	*“My son refused to wear it because he felt the peer pressure… and he didn’t want to be the only one wearing a mask. It wasn’t even spoken or verbal. It was just, he didn’t want to be the uncool one wearing a mask.”* (Latinx English)
	Masculinity	*“I’ve noticed it’s mostly men who seem to be against wanting to wear masks. I don’t know if it’s a pride type of thing, or like I’m too cool for this or whatever.”* (Rural white)
	Political affiliation	*“I mean, honestly, at this point, like the people that I work with, the only person they’re going to listen to is Trump. So unless Trump tells them to wear a mask, they’re not gonna wear a mask.”* (Young adult white)
	Individual rights and freedom	*“I like you know having the choice to do it. And having the choice, I’m still going to do what I can to protect people. But it being required, I don’t necessarily like that.”* (Rural white)

**Table 4:** Insights for messaging strategies informed by focus groups with North Carolina residents.

Category	Insight
General areas of focus	Focus on protection as a motivator for the use of face coverings
	Promote and normalize the use of face coverings in social and work settings
	Emphasize face covering requirements
	Encourage not only the use but the correct use of face coverings
Message content	Harness the power of personal experiences with COVID-19
	Support messages with data
	Clarify mixed messages and answer nuanced questions about the use of face coverings
	Balance attention to high-risk groups with inadvertently promoting invincibility
	Focus on recovery, but do not promise a return back to normal
Message tone	Frame messages as positive and encouraging
	Promote unity and diversity
	Use language that resonates with specific communities

## Data Availability

The data presented in this study are available on request from the corresponding author. The data are not publicly available due to the consent provided by participants on the use of confidential data.

## Supplementary Materials

The following are available online at https://www.mdpi.com/1660-4601/17/24/9298/s1, Focus Group Discussion Guide, Table S1: Sample face covering messages tested in focus groups with North Carolina residents.
