# Supplementary material for: Motivations and Barriers for the Use of Face Coverings during the COVID-19 Pandemic: Messaging Insights from Focus Groups
Source: Int J Environ Res Public Health. 2020 Dec 12;17(24):9298. doi: 10.3390/ijerph17249298 (PMC7763909; doi:10.3390/ijerph17249298)
Supplement: Supplementary file 1 [file ijerph-17-09298-s001.pdf]

Focus Group Discussion Guide

1. In what situations, if any, do you wear face coverings?
    a. *Probe for specific situations: (1) when running errands, (2) when visiting with friends and family, (3) when at work outside the home*
    b. *What type of face covering do you wear in these situations?*
    c. *How frequently do you wear it in this situation?*
    d. *Why do you wear it in this situation?*
    e. *How do you feel about wearing a face covering?*
2. What are some of the reasons you wear a face covering?
    a. *How does the behavior of people around you affect your decision to wear a face covering?*
    b. *What about enforcement of requirements to wear face coverings?*
    c. *What have you heard about whether face coverings can prevent people from getting COVID-19?*
3. In what situations do you <u>not</u> usually wear face coverings?
    a. *Probe for specific situations: (1) when running errands, (2) when visiting with friends and family, (3) when at work outside the home*
4. What are some of the reasons you do <u>not</u> wear a face covering in these situations?
5. What do you think are some of the reasons that <u>other people</u> in your community, including your family and friends, don't wear face coverings?
    a. *How do your friends or family react if you wear a face covering around them?*
6. Can you think of any messages that were especially memorable or meaningful for encouraging the use of face coverings?
    a. *Whose opinions about face coverings are most important to you?*
    b. *Who do you turn to for advice about face coverings?*
7. I'm going to display a few messages about face coverings and I would like to hear your opinions about them. What do you think about this message?
    a. *What do you like about this message? What do you dislike about this message?*
    b. *How could this message be improved?*
    c. *Does this message make you want to use face coverings?*
    d. *Who do you think this message is written for?*
    e. *What image comes to mind when you hear this message?*

Table S1. Sample face covering messages tested in focus groups with North Carolina residents

Theme	Sample Messages
**1**	Mask on. To carry on.<br>Mask up. To open up.<br>Cover your face. Keep some space. #StayStrongNC
**2**	Please wear a face covering to speed up NC’s recovery.<br>Vice President Mike Pence believes wearing a face covering is a good idea. Follow his lead.
**3**	Friends don’t let friends go out without a mask.<br>You wouldn't wish it on your enemy, so why risk giving it to your friend? Please wear a face covering around others.<br>Make sure your memories don't turn into regrets. Wear a face covering around others.
4	Protect your grandmother, your neighbor with cancer, and your best friend with asthma. Use face coverings when out in public.<br>Help keep loved ones safe. Use face coverings when out in public.
